# Iodine deficiency in pregnancy

**DOI:** 10.1186/1824-7288-40-S1-A16

**Published:** 2014-08-11

**Authors:** Alessandra Cassio

**Affiliations:** 1 Department of Medical and Surgical Sciences, Pediatric Unit, Program of Pediatric Endocrinology, University of Bologna, Bologna, Italy

## 

Iodine is an essential micronutrient for thyroid hormone synthesis and thus is essential for the normal progression of pregnancy and for the fetus neuropsychological development. Iodine requirements increase during pregnancy for physiological changes in iodine metabolism (increased renal iodine losses, iodine transfer from maternal circulation to the fetoplacental unit and fetal iodine needs for thyroid hormone production).

Therefore the iodine intake should be increased by about 50% during pregnancy and lactation and the daily dose recommended by the WHO is at least 250 µg / day corresponding to a Urinary Iodine Excretion ( UIE ) range of 150-249 µg /L. Iodine supplementation is recommended in all pregnant women , even in iodine sufficient countries or in areas where universal salt iodization has been achieved (iodized salt consumed in more than 90% of households). These measures, in fact , ensure the daily iodine intake of 150 µg / day recommended in all women of childbearing age but they are not sufficient to support the increased requirements imposed by pregnancy. Recently, even in developed countries, such as UK, USA, and Australia , moderate iodine deficiency has re-emerged as an important public health concern , likely due to a change in eating habits of the population.

There are recent data that highlight how even mild degrees of iodine deficiency may adversely affect the progression of the pregnancy or the neurocognitive outcome in children. It should be emphasized that national programs of health policy must provide epidemiological surveillance measures to ensure the maintenance of iodine sufficiency over time.

Because the fetal thyroid gland may be particularly sensitive to the inhibitory effect of high iodine concentrations , possible negative effects of maternal iodine supplementation during pregnancy have been hypothesized. The currently available data, however, appear to indicate a relatively low risk of these effects against the benefits derived from the adequate transfer of maternal T4 to the fetus in conditions of iodine sufficiency especially in the first trimester of gestation.

